# A Practical Treatise on Diseases of Children

**Published:** 1854-01

**Authors:** 


*A Practical Treatise on Diseases of Children.* By D. Francis Condie, M. D., etc., fourth edition revised and improved. Philadelphia: Blanchard & Lea, 1854.

Dr. CONDIE's work has been in the hands of the profession for some time, and the fact that a fourth edition has been called for, is sufficient evidence that its value is appreciated.

The present edition is fully equal in mechanical execution to any which has preceded it.

For sale by D. B. Cooke & Co., 135 Lake st. J.

